# How did COVID-19 pandemic affect the older adults’ needs for robot technologies in Japan?: comparison of participatory design workshops during *versus* after the COVID-19 pandemic

**DOI:** 10.3389/frobt.2024.1363243

**Published:** 2024-06-04

**Authors:** Takanori Komatsu, Marlena R. Fraune, Katherine M. Tsui, Shogo Suda, Mizuki Kobayashi

**Affiliations:** ^1^ Department of Frontier Media Science, Meiji University, Tokyo, Japan; ^2^ Department of Psychology, New Mexico State University, Las Cruces, NM, United States; ^3^ Toyota Research Institute, Cambridge, MA, United States

**Keywords:** social robots, participatory design, co-design, older adults, social challenges

## Abstract

Social technology can improve the quality of social lives of older adults (OAs) and mitigate negative mental and physical health outcomes. When people engage with technology, they can do so to stimulate social interaction (stimulation hypothesis) or disengage from their real world (disengagement hypothesis), according to Nowland et al.‘s model of the relationship between social Internet use and loneliness. External events, such as large periods of social isolation like during the COVID-19 pandemic, can also affect whether people use technology in line with the stimulation or disengagement hypothesis. We examined how the COVID-19 pandemic affected the social challenges OAs faced and their expectations for robot technology to solve their challenges. We conducted two participatory design (PD) workshops with OAs during and after the COVID-19 pandemic. During the pandemic, OAs’ primary concern was distanced communication with family members, with a prevalent desire to assist them through technology. They also wanted to share experiences socially, as such OA’s attitude toward technology could be explained mostly by the stimulation hypothesis. However, after COVID-19 the pandemic, their focus shifted towards their own wellbeing. Social isolation and loneliness were already significant issues for OAs, and these were exacerbated by the COVID-19 pandemic. Therefore, such OAs’ attitudes toward technology after the pandemic could be explained mostly by the disengagement hypothesis. This clearly reflect the OA’s current situation that they have been getting further digitally excluded due to rapid technological development during the pandemic. Both during and after the pandemic, OAs found it important to have technologies that were easy to use, which would reduce their digital exclusion. After the pandemic, we found this especially in relation to newly developed technologies meant to help people keep at a distance. To effectively integrate these technologies and avoid excluding large parts of the population, society must address the social challenges faced by OAs.

## 1 Introduction

The COVID-19 pandemic brought drastic changes to humanity’s way of daily life. Because of the extremely high infectivity of COVID-19 virus, people were required to maintain social distance, and refrain from activities normally taken for granted, like going to school or work, eating or drinking out, traveling, and gathering with close relatives. This resulted in the loss of opportunities to meet new people and maintain old friendships, a loss of social mobility, fragmentation of intergroup interaction and extreme social isolation (Buecker and Horstmann, 2021; Donizzetti and Lagacé, 2022; Rodrigues et al., 2022; Murayama et al., 2023). Especially, the COVID-19 had a higher mortality rate in older adults (age 50+; OAs) (Morrell et al., 2000; Sum et al., 2008; Gao et al., 2015; Itoh et al., 2021) compared to the other age groups. To save lives, health professionals recommended that OAs avoid contact, even with close friends and family members. Thus, OAs were overexposed to the risk of adverse mental health outcomes, including a complete loss of opportunities for daily and traditional social interaction. Such strong restrictions of in-person activities made OAs being socially isolated or feel loneliness (Armitage and Nellums, 2020; Tyrrell and Williams, 2020).

The digital technology revolution coincided with the COVID-19 pandemic (Almaiah et al., 2020; Papagiannidis et al., 2020; Waizenegger et al., 2020; Abu Talib et al., 2021; Kniffin et al., 2021; Ben-Zvi and Luftman, 2022). Various information technologies were introduced at a rapid pace into people’s everyday lives in order to maintain normal life as much as possible, such as remote learning and employment. This included videoconferencing for online teaching and working, electronic money for non-contact money exchange, self-checkouts and home delivery services for shopping without human contact are typical examples. Thus, viewing the COVID-19 pandemic from a social perspective, it caused a breakdown everywhere in society by forcing individuals to live with extremely restricted behavior; however, viewing it from a technological perspective, it enabled the rapid introduction of digital transformation. In terms of the relationship between social isolation and information technology use, Nowland et al. (2018) proposed the stimulation and disengagement hypotheses; that is, the former one is that people use information technology to enhance social relationships which decrease loneliness, the latter is that people use information technology to escape from their own lives, which increase loneliness. Therefore, to clarify how the large periods of social isolation like during the COVID-19 pandemic affect whether the OAs use technology in line with the stimulation or disengagement hypothesis is quite meaningful to understand the social challenges faced by OAs.

To comprehend the OA’s attitude toward the technologies, we utilized the participatory design (PD), which is a design methodology in which end-users are involved in the design process to ensure that the final product meets the needs of them (Lee et al., 2017). In our PD, the participants were required to consider what social problems they are facing and what kinds of robotic technology would resolve these problems. This procedure can allow participants to effectively reflect their issues specific to the pandemic and accurately grasp their current situation. We then conducted the PD workshops in June 2023 when the COVID-19 pandemic has ceased (about 1 month after the US declared that the COVID-19 pandemic has ceased^1^) so people were returning to their pre-pandemic lifestyle. We then compared the results of our last August 2021 workshop (during the pandemic) (Fraune et al., 2022) and this June 2023 workshop (after the pandemic), and then examined how the COVID-19 pandemic affected the social challenges OAs faced and their expectations for robot technology to solve their challenges changed from during to after the pandemic. We finally considered how the results of PD workshops during and after the pandemic can be explained by the Nowland’s stimulation or disengagement hypotheses.

Here, the following two research questions would be a guide of this paper.

• RQ1: How did OAs’ needs for robot technology change from during to after the pandemic?
• RQ2: How were OAs’ attitudes toward such technology during and after the pandemic explained by the stimulation and/or disengagement hypothesis?

## 2 Related works

### 2.1 Social isolation and loneliness

Social isolation, defined as low quantity and quality of social and emotional connections (Shankar et al., 2011), increases loneliness and decreases physical and mental health (Weiss, 1973; Van Baarsen et al., 2001; Wang et al., 2003; Tomaka et al., 2006). Loneliness, a negative emotional state due to a discrepancy between the social relationships people wish to have and the ones they perceive they actually have (Heinrich and Gullone, 2006), relates to increased social anxiety, risk of depression, suicidal ideation, and reduced cognitive functioning (Heinrich and Gullone, 2006; Hawkley and Cacioppo, 2010). Loneliness also closely relates to negative health conditions like cardiovascular disease (e.g., high blood pressure, high cholesterol) and risky life-threatening habits (e.g., smoking, lack of exercise) (Hawkley and Cacioppo, 2010; Shankar et al., 2011).

Social isolation and loneliness are especially concerns for OAs and are also prominent issues in Japan, which is categorized as a super-aged society (Yasunaga et al., 2017; Mitsutake et al., 2018)^2^. Muramatsu and Akiyama reports that the OAs in Japan are more socially isolated than those in France, Germany, South Korea and the US (Muramatsu and Akiyama, 2011). The mental and physical impacts of social isolation and loneliness in OAs is strongly associated with more frequent medical visits, earlier onset of cognitive decline and Alzheimer’s disease, and risk of all-cause mortality (Wilson et al., 2007; Gerst-Emerson and Jayawardhana, 2015; Boss et al., 2016; Beller and Wagner, 2018).

### 2.2 COVID-19 and societies

COVID-19 (Coronavirus disease 2019) was an epidemic disease caused by the virus severe acute respiratory syndrome coronavirus 2 (SARS-CoV-2), which originated in Wuhan, China in December 2019 and subsequently spread globally. By September 2023, more than 690 million people had been infected (Worldometer, 2022). In response to the rapid spread of the virus, governments around the world ordered their citizens to observe various degrees of physical distancing (i.e., maintaining physical distance between an individual and people not living in the same household), from restrictions on international travel to mandatory stay-at-home orders (Giallonardo et al., 2020; González-Rodríguez and Labad, 2020; Moreland et al., 2020). Such strong restrictions of in-person activities increased the worldwide prevalence of mental health conditions like anxiety and depression (Lytridis et al., 2020; Saladino et al., 2020; Sikali, 2020) and especially loneliness in OAs (Buecker and Horstmann, 2021; Donizzetti and Lagacé, 2022; Rodrigues et al., 2022; Murayama et al., 2023). By September 2023, the COVID-19 pandemic had subsided in many countries, but there are currently limited studies reporting how the end of the COVID-19 pandemic affect the OAs.

Researchers have conducted studies on the impact of COVID-19 on various aspects of society in Japan (Handler and Kawaminami, 2023; Kobayashi, 2023; Tanikaga et al., 2023). One study investigated how and to what extent pandemic-induced novel telecommuting affected employees’ travel, activities, and residence locations and explored their expectations of post-pandemic life (Liang et al., 2023). Another (Arai et al., 2023) showed that opportunities to participate in society were disproportionately reduced for people with disabilities during the COVID-19 pandemic. Tani et al. (2023) found that the flourishing, which is conceptualized as “a state in which all aspects of a person’s life are good,” declined during the pandemic, especially for men and lower-educated people.

The COVID-19 pandemic led to the rapid introduction of information technologies into everyday life to maintain normal life as much as possible, like education and employment (Papagiannidis et al., 2020; Ben-Zvi and Luftman, 2022). Many studies investigating technology use during the pandemic focused on educational aspects, like how online learning was constructed and how it benefited learning styles (Almaiah et al., 2020; Abu Talib et al., 2021), and on employment aspects, like how online employment facilitates team work performance (Waizenegger et al., 2020; Kniffin et al., 2021). A meta-analysis of nearly 300 articles about the relationship between humans and information technologies during the COVID-19 pandemic found that information technologies were mainly used in specific domains like education and employment, and also used in healthcare and daily use (Vargo et al., 2021). A few articles report how OAs have used these technologies for healthcare and family interactions (Elbaz et al., 2021; Racin et al., 2023; Tang et al., 2023).

### 2.3 Technology and people

When technology develops, especially quickly, it is important to ask who the technology is being developed for and how well it meets their needs. As scholars of design justice point out, people create technology based on existing biases, which tends to favor groups in the majority (Costanza-Chock, 2020). This, and other situational factors, create digital exclusion of certain groups of people. Digital exclusion (Age UK, 2018) occurs when people do not have access to technology, the skills to use technology, or cannot benefit from the outcomes of the technology (Blank and Groselj, 2014; Government Digital Service, 2014; van Deursen and Helsper, 2015; Scheerder et al., 2017). People were typically considered digitally excluded include those who are people in a minority ethnic group, people with disabilities, and OAs, the last of which we study in this paper. Thus, with new technology developing especially rapidly, it can place heavier burdens on people who are already digitally excluded because they not only need to adapt to a new technology like everyone else, but they need to adapt to a technology that does not suit them.

Using technology to one’s social benefit is not only a matter of feeling comfortable with the technology, but using it in a way that leads to more social connection. In Noland et al.‘s model (Nowland et al., 2018) of the relationship between social Internet use is loneliness, they propose to hypotheses that are not mutually exclusive: Evidence for the stimulation hypothesis shows that people can use information technology to enhance social relationships, which decreases loneliness (Sum et al., 2008; Elliot et al., 2013; Lee et al., 2020). Evidence for the disengagement hypothesis shows that people can also use information technology to escape from their own lives, which leads to increased loneliness (Kraut et al., 1998; Nie, 2001; Nie et al., 2002; Kato et al., 2019). Likewise, if people have access to technology, but cannot use it well, they do not benefit from its use (Chen and Schulz, 2016; Chopik, 2016). Research on OAs tends to support the stimulation hypothesis. Social technologies can assist them in maintaining contact, especially with existing social networks (Cotten et al., 2012; Wilson et al., 2021). Social technology can also reduce social isolation in OAs (Bruck, 2002; Clark, 2002). More frequent social technology use related to fewer mental and physical health problems and higher self-perceptions of wellbeing in OAs (Chopik, 2016). During the pandemic, OAs had the possibility of connecting with others through social technology, but they only gained these benefits if they had high affinity for technology (Fraune et al., 2023a). Recent work shows that digital exclusion acts as a barrier to obtaining benefits from stimulation via social technology (Ling et al., 2023).

### 2.4 Participatory design

Participatory design (PD, or co-design) is a design methodology in which end-users and people other than traditional researchers and designers are involved in the design process to ensure that the final product meets the needs of users (Lee et al., 2017). Through PD, participants first identify their own needs and desires, and then co-design a technical solution to meet those needs. PD workshops are qualitative research and tend to have small numbers of participants because of the long time required for conducting workshops (Gliner et al., 2002; Mason, 2010; Amrhein et al., 2019), however PD is valuable for helping researchers to understand perspectives of diverse users and stakeholders in different contexts (DiSalvo et al., 2008; Šabanović et al., 2015; Georgiou et al., 2020).

OAs are often stereotyped as less willing to use new technologies, so they have not been recognized as end-users of many previous technology developments. However, PD workshops are particularly valuable and empowering for OAs (Lee and Šabanović, 2014; Laura Ramírez Galleguillos and Coşkun, 2020; Fraune et al., 2022). Therefore, this methodology has attracted a lot of attentions in a research area of human-robot interaction especially how to design the social robots for OAs (Eftring and Frennert, 2016; Thunberg and Ziemke, 2021; Ostrowski et al., 2021a; b, 2022; Fraune et al., 2022; Alhouli et al., 2023; Stegner et al., 2023). For example, Ostrowski et al. (2021a) proposed a year-long co-design methodology leveraging convergent and divergent design activities to empower OAs in the technology design process with researchers based on seven sessions in a co-design project of home social robots with 28 OAs. This long-term co-design principle was deliberately and carefully proposed so this strongly supports and calls for respectful and responsible co-design with communities who may, in the future, interact and live with robots. Fraune et al. (2022) proposed an approach to participatory design of future technologies that spends 2/3 of the PD sessions asking participants about their own life experiences as a foundation. This grounds the conversation in reality, creates rapport among the participants, and engages them in creative critical thinking. We already conducted the PD workshops during the COVID-19 pandemic (August 2021) by means of the above Fraune’s methodology (Fraune et al., 2022), so we also utilized this in PD workshops after the pandemic (June 2023).

## 3 Methods

### 3.1 Design teams

We recruited in 12 participants who satisfied the following requirements; (1) 50+ years of age, (2) able to use video conferencing software, (3) a resident of Japan and spoke Japanese, and (4) no prior experience participating in PD activities beforehand. Participants (N = 12; age M = 57.9, range = 50–65, five men and 7 women) were recruited through a human resource dispatching company and compensated 7,000 JPY per participant. There was no participation overlap between our PD workshops in August 2021 and June 2023. The term “older adult” is typically used to describe people age 65 years or older, which is the ideal target population of our research. Some previous work using participatory design in the HRI literature has also used older adults aged below 60 (Šabanović et al., 2015) and similarly, there is evidence of wider age bands regarding research on smartphones (Gao et al., 2015), internet use (Morrell et al., 2000; Sum et al., 2008), and care technologies for activities of daily living (Itoh et al., 2021). These papers used the term “Older adults” as over 40–55, so our criteria “OA is 50+” is quite reasonable.

The PD sessions remained virtual (Feil-Seifer et al., 2020). Each workshop included two facilitators and three participants. Facilitators had a background in information technology and research, and participants did not. During sessions, one facilitator led the discussion, posed all questions and brainstorming prompts, and moderated the discussion. One note-taker paraphrased participant comments and themes of the discussion on a shared screen throughout the workshop.

### 3.2 Procedure

The PD workshops took place on June 17 and 18, 2023. Sessions occurred via Zoom online video chat and were video recorded. Researchers mailed participants study supplies (post-it notes, markers) and printed ethics consent form, and they mailed the signed form to us before the workshop. Session lasted approximately 75 min, consisting of three rounds, each focused on a different main theme. Rounds began with a 5-min brainstorming phase, then used a ‘round-robin’ discussion format, with the facilitator allowing each participant the opportunity to share an initial idea one at a time before opening the floor to a more free-form discussion. This ensured that each participant contributed their ideas during the early stages of each round, so that the following discussion was informed by the opinions of all present. Through all rounds, the note-taker paraphrased participant comments and main ideas on Google Slides, using it as a shared ‘digital whiteboard’ to provide a common reference point for continued discussion.

Before the round 1, participants viewed a video of current commercial robots to ensure all participants had some understanding of current robotic technology’s capability, as opposed to drawing from movies (Sundar et al., 2016).

Round 1: Participants discussed the technologies they currently use to communicate with others. The facilitator asked both what they liked about social experiences facilitated by technology, and what aspects of their technology-mediated interaction were missing or altered when compared to in-person interactions.

Round 2: Participants reviewed social challenges they currently faced, such as keeping up with old friends, making new connections, or socializing at large gatherings. The facilitator welcomed participants to share both new challenges specific to the unique social circumstances of the time and general challenges that existed before social distancing norms of COVID-19 pandemic.

Round 3: Participants brainstormed ideas for robots to help solve one of the social challenges discussed in Round 2. The facilitator encouraged participants to focus on ideas for a robot they would personally want to own and use and would be technically feasible within the next 3 years. After initial brainstorming and discussing their individual ideas, participants engaged in iterative design by picking one idea discussed (either their own or another participant’s) to improve upon, or add to, in a subsequent 3-min brainstorm session. Another round-robin discussion followed this second brainstorming session. When appropriate, the facilitator re-focused the discussion or posed high-level questions, such as “What problem is the robot solving?” and “What might be some challenges of that idea?” Finally, the facilitator asked participants to create a list of their five favorite robot features discussed in this round.

### 3.3 Analysis

We transcribed video-recordings of each workshop session. Researchers then analyzed and coded these transcripts along common themes. Themes were derived from open and axial coding (Glaser et al., 1968) which was translated in Japanese. Specifically, two experimenters independently worked to check the participants’ statements and to select the appropriate coding categories for each statement. Interrater agreement between these two experimenters in all four PD sessions ranged from moderate to strong (IRRs > 0.65) (Miles and Huberman, 1994). Note that the previous workshops in 2021 mainly focused on the comparisons between Japan and the US, whereas this workshop was conducted only in Japan, so we compared the Japanese data during and after the pandemic.

### 3.4 Results of PD study during the COVID-19 pandemic

In order to compare the results of the PD workshops during and after COVID-19 pandemic, we summarize below the results of PD studies conducted in August 2021, in Japan (during COVID-19 pandemic). Participants (N = 12; age M = 54.42, range = 50–63, 6 men and 6 women) were recruited through a human resource dispatching company and compensated 7,000 JPY per participant. For the detailed results, see Fraune et al. (2022).

#### 3.4.1 Round 1: current technology use

LINE^3^ was the most frequently used technology. While the participants felt that communicating with others through the technology without worrying about location or time was an advantage, they also thought that the lack of the information they could obtain through the technology was a disadvantage compared to face-to-face communication.

#### 3.4.2 Round 2: current social challenges

Regarding existing social relationships, OAs lost most opportunities to anyone outside their households, including family members who live apart. Therefore, their most significant difficulties were with keeping in contact with these family members, especially their parents who were not good at using current technologies.

Regarding connecting with new people, participants found it difficult to create new connections with the others via only online technology, due to difficulties discussed in Round 1, about the limited information available via online technology.

#### 3.4.3 Round 3: robot design concepts

Participants’ final robot design concepts followed three themes: (1) pet robot, (2) sharing experiences, and (3) easy operation. Pet robot: Many participants wanted to place a pet robot in their parents’ house to provide them with a social companion when they were unavailable to visit. Participants expected pet robots to be useful in relieving loneliness of parents who are apart and in communicating and dealing with problems when they occur. Sharing experience: Although there were no concrete discussions about how the robots can resolve these issues, half of the participants said that they felt huge challenges of sharing experiences (e.g., sharing pictures from a recent trip). Easy operation: Many participants required help from tech-savvy people when facing new devices or applications. They wanted robots with easy operation because they wanted to place these robots at the parents’ house on the behalf of the participants.

To wrap up the PD workshop during the COVID-19 pandemic, participants’ most significant social difficulties were in maintaining existing relationships, such as with family members, including their parents who live apart. They wanted to use robotic technologies to resolve this issue; specifically, they wanted to place an easily-operated pet robot that could mitigate their parents’ loneliness and could watch them on the behalf of the participants in case they have a problem (e.g., falling down due to unexpected injury or illness).

## 4 Results

### 4.1 Round 1: current technology use

In Round 1, the participants discussed the technologies they currently use to communicate with others, and the advantages and disadvantages of those technologies. The most common application was LINE, followed by videoconferencing applications like Zoom^4^ and Microsoft Teams^5^. Several participants used ChatGPT^6^ to communicate with AI systems. They mainly used these applications on smartphones rather than PCs^7^.

#### 4.1.1 Benefits of current technology

Participants enjoyed that they could communicate with others regardless of where they were. “I don’t need to travel all the way to meetings and conferences, so I can use my time more effectively (P3).” “Since these technologies are portable, I can take them with me when I go out and immediately get contact the other person wherever and whenever I want (P10). “They enjoyed using one device or application for multiple purposes: “These applications are in my smartphone, so I can carry these with me easily (P2),” “(LINE) can be used not only for text chatting but also for video and voice calls (P1).”

They also enjoyed elements of the technology that made social interaction via it more seamless. “(Text chatting) is easier and smoother than e-mail (P7),” “ (LINE) shows already read remark when the other person reads my message, so their responses are quick (P1).” Some also enjoyed that it was not a financial burden, liking the “Free video calling (P10).”

#### 4.1.2 Drawbacks of current technology

The main drawback participants discussed was uncertainty about nuance and communication due to the limited information they could gain from the technology. For example, P1 said, “In video calls such as Zoom, I don’t feel like I am looking at the other person eye-to-eye, so I worry about whether my facial expressions are properly conveyed to the other person.” Similarly, P11 said, “Online video call cannot convey detailed nuances to the others,” and P3 said, “I cannot understand the mood of the situation because I cannot see the other person’s facial expressions in detail.” P12 pointed out the disadvantages of text communication, like “I am worried that I cannot convey my feelings well using only text or letters, which can lead to miscommunication and misunderstanding from the others.”

Overall Round 1, while most participants mentioned that the current technologies communicating with others have advantages that can beyond their constraints of place and time, they also mentioned that these have some disadvantage in that the amount of information they could obtain was less than in face-to-face communication.

### 4.2 Round 2: Current social challenges

In Round 2, participants discussed social issues about human relationships that they themselves face, especially about maintaining existing social relationships and connecting with new people.

#### 4.2.1 Challenges maintaining existing social relationships

Participants discussed the difficulties and problems they experience in maintaining existing social relationships. Regarding technology for communication, which was the focus of the previous Round 1, P7 and P9 responded, “when I want a reply from the other person, but I don’t get a reply, I get frustrated or anxious,” and P9 responded, “I can easily join some groups on the Internet, but it is difficult to get out of them.” P11 indicated that she worried about problems caused by miscommunication or misunderstanding of the contents of e-mails.

As general difficulties not related to technology, P2 and P3 were careful in their interactions with people younger than themselves, and P1 and P10 said that it was important to maintain appropriate distance from others (e.g., switching between public and private) was important for maintaining appropriate relationships with them.

#### 4.2.2 Challenges connecting with new people

Participants discussed perceived difficulties and problems in connecting with new people, related to the lack of information compared to in face-to-face environments, as discussed in Round 1. P6 responded, “When communicating with people I am meeting for the first time, it is more difficult to convey my personality and atmosphere via online than in person.”

In addition, although it has become easier to exchange contact information such as via SMS, some participants had general anxiety about relationship-building. P7 worried about how to build relationships from there, and P9 was concerned about what the other person thinks about them. P11 usually feels nervous because he believes he is not good at interacting with others, and P12 wondered how much information about himself he should disclose to others to avoid oversharing them.

### 4.3 Round 3: robot design concepts

In Round 3, participants discussed ideas for designing a robot that can solve problems discussed in Rounds 1 and 2 and they picked the top five features they would want for a robot. Participants’ final robot design concepts surrounded three themes: 1) Interactive robot, 2) Proxy robot, and 3) Assistant robot.

#### 4.3.1 Interactive robot

The most frequently mentioned robot concept was the interactive function. In all sessions, participants discussed interactions and conversations with the robot, with 10 out of 12 participants speaking on this topic. Many participants indicated that they themselves would like to be able to interact with these robots to relieve their loneliness, to feel comfort, or to have someone to talk to. For example, P10 said, “I would like to have the robot that can talk to me. This robot would be a beloved friend if this robot shows the personalized behavior based on my input data, my own preference and personality.” P5 also stated, “I think it would be good in the future to have a robot that has a function like ChatGPT: that the robot can answer various kinds of questions we have,”

#### 4.3.2 Proxy robot

There were also active ideas for robot concept that the robot performs various tasks on behalf of the participants. For example, P7 focused on complicated procedures of on-line application for government offices, saying, “When I search on the Internet, I could find contact information and phone numbers of the government office, but after a while, I could not find the phone numbers and only can inquire by e-mail, so I am wondering where to contact exactly … ” P10 was also concerned about the replacement of technology in all aspects of daily life, saying, “At the unmanned checkouts in supermarket, it is a bit difficult for elderly people to use this. If possible, I want to use the in-person checkouts. If there is a question, I know that people can ask questions via the monitor at this checkout, but I often see some people having trouble at there.” P8, who felt that he was not able to make good use of the functions implemented in smartphones, added, “Even though I look at map apps, I often get lost on my way, especially when I am in a bit of a hurry and have a fixed time. It would be nice if there was an alternative that could guide me in such situations.”

#### 4.3.3 Assistant robot

There was also active discussion about a robot that does not take over all the works like a proxy robot but that assists users. For example, P1 remarked, “If there is a robot that manages schedules and appointments and tells me what to do, I think we can maintain a good relationship because it will not bother other people if I accidentally forget my schedule or something.” Other opinions were also expressed, such as a robot that can think about the text of e-mails together with us (P10, P12), and a robot that can subtitle the audio of video calls (P9).

### 4.4 Common features of PD sessions during and after pandemic

Across the two PD studies in August 2021 (during the pandemic) and in June 2023 (after the pandemic), we found similarities in responses in Rounds 1 about current technology use and Round 2 about connecting with new people.

#### 4.4.1 Round 1

Both during and after the pandemic, Round 1 was very similar. The most commonly-used technology was LINE. Participants appreciated the advantage of social technology was that there were no restrictions on time and place. The main drawback they felt was that they could not obtain a lot of information that face-to-face communication can easily (e.g., body posture).

#### 4.4.2 Round 2

Both during and after the pandemic, Round 2 included similar discussions about the challenges of connecting with new people. Because of difficulties discussed in Round 1 about the lack of information during online interaction, they had trouble connecting new people online.

While technology that connects people online is effective for OAs in maintaining the existing relationships, it is not well suited for them in connecting with new people. Indeed, higher levels of affinity for technology during the pandemic related to higher perceived group cohesion with new groups, but not existing groups (Fraune et al., 2023b). Conversely, younger generations are very active building new relationships through the use of online technology, such as dating apps, so this may cause a generation gap in the adoption of such technology (Gibson, 2021; Joyce et al., 2022).

### 4.5 Different features of PD sessions during and after pandemic

Although Round 1 and challenges of connecting with new people in Round 2 were similar during and after the pandemic, there were the completely different discussions about challenges of maintaining the existing relationship (Round 2) and about preferred robot design concepts (Round 3).

#### 4.5.1 Round 2

In Round 2, participants had different experiences with challenges of maintaining the existing relationship during and after the pandemic. During the pandemic, because of restrictions against meeting others face-to-face, participants had difficulty interacting and communicating with family members, especially those who lived far from them. Therefore, in the PD workshops during the pandemic, interaction and communication with elderly parents who live apart was a particularly serious problem. Conversely, after the pandemic, with the restrictions removed, only a few participants had concerns about communicating with family members. Few participants used the terms “COVID-19” or “parents who live apart” in the workshops after the pandemic. Instead, participants’ current challenges were general anxieties about ordinary social life that probably existed before the pandemic related to socially integrating OAs (Muramatsu and Akiyama, 2011), like “how to interact with or how to keep comfortable distance to younger generations in their office,” or “My SMS message was already read by the others, but still there is no response.”

#### 4.5.2 Round 3

In Round 3, there was a vast difference in robot concepts that might resolve present social challenges. The final three robot design concepts in the PD workshops during the pandemic were *Pet robot, Sharing experience,* and *Easy operation,* while in the workshops after the pandemic, they were *Interaction robot, Proxy robot,* and *Assistant robot.* Thus, participants in both PD workshops suggested robot solutions from different perspectives.

## 5 Discussion

### 5.1 Stimulation/disengagement hypotheses and PD workshops

Here, we focused on the different features in “Round 3” of PD sessions during and after the pandemic, and discussed these in more depth in terms of the stimulation and disengagement hypotheses Nowland et al. (2018). Specifically, we considered which robot concepts can be explained by stimulation or disengagement hypotheses. Table 1 showed that the top three concepts of robots in round 3 and the following hypotheses (stimulation or disengagement) during and after pandemic.

**TABLE 1:** Top three concepts of robots in round 3 and following hypotheses of PD workshops during and after the pandemic.

During-pandemic		After-pandemic	
Top 3 concepts	Hypotheses	Top 3 concepts	Hypotheses
Pet robot	Stimulation/Disengagement	Interactive robot	Disengagement
Sharing Experience	Stimulation	Proxy robot	Disengagement
Easy operation	Stimulation	Assistant robot	Stimulation/Disengagement

In PD during the pandemic, most participants had serious concerns and difficulties communicating with family members or elderly parents, especially those who live apart from them. Perhaps in reaction to this new distance, participants’ responses during the pandemic relate most strongly to the stimulation hypothesis and reducing digital exclusion. The theme of wanting to *share experiences* relates strongly to the stimulation hypothesis, helping people connect socially with others. *Ease of operation* relates to wanting to reduce digital exclusion for themselves and their family members. This suggests that during the pandemic, OAs were trying to maintain social connection, following the stimulation hypothesis. The desire for *pet robots* for parents, however, links to both the stimulation and disengagement hypotheses. It relates to the stimulation hypothesis because people wanted the robot to create a social connection with their parents. However, it relates to the disengagement hypothesis because this robot would engage in social interactions, rather than participants engaging in them. The theme of *pet robot,* in which these two hypotheses of different nature are compatible, may reflect the uniqueness of robot technology in Japanese society, because it was not observed in PD sessions conducted at the same time in the US (Fraune et al., 2022).

In PD after the pandemic, participants had concerns not about family members who live apart but about themselves, and their responses relate mostly to the disengagement hypothesis and increasing digital exclusion. Although we instructed them “to brainstorm ideas for robots to help solve one of the social challenges discussed in Round 2,” their discussions shifted from the challenges about the communication between persons to the one about the relationship between themselves and societies, reflecting their concerns about their position or status in their societies. For example, in PD after the pandemic, participants discussed *interactive robots*. However, the reason they needed these robots was not for other people (like *pet robots* for their parents), but for relieving the OA participants’ own loneliness. This relates to the disengagement hypothesis because they want to interact with the robot rather than with other people through the robots. The themes of the *proxy robots* and *assistant robots* were to dispel or resolve their own anxiety about the latest technologies such as online applications at government offices or self-checkouts, which are rapidly increasing (Duarte et al., 2022). We speculate that the rapid digital transformation made the OAs uneasy and anxious for these useful technologies. Therefore, this would be the reason why they need the proxy robots facing to these technologies on their behalf. These themes (*proxy robots* and *assistant robots*) are similar to the *ease of use* theme from the during-pandemic PD sessions; however, the participants after the pandemic wanted ease of use from themselves, whereas during-pandemic participants wanted “ease of use” for their parents. This relates to the disengagement hypothesis because they wanted the robot to work in their society instead of them. However, in case of *assistant robot*, the participants wanted the robot to help with their social activities (e.g., organizing the participants’ schedule, or checking the incoming messages). This also relates to the stimulation hypothesis because this facilitates their engagement to their society. Here, the theme of *assistant robot* was explained by both stimulation and disengagement hypotheses, like *pet robot* was during the pandemic, so the required functions for *assistant robot* and *pet robot* seems to be similar–that is, not only taking care of or helping somebody (elder parents or themselves) but also connecting to the others (children or their society).

Overall, although the COVID-19 pandemic led to the rapid adoption of various kinds of useful systems that can allow the same functional processing as before the pandemic without human contact, such rapid shift to digital transformation (DX) seemed to made OAs uncomfortable. Our two PD workshops during and after the pandemic captured the OA’s concerns and difficulties with current social challenges. Analyzing their difficulties from the viewpoint of the stimulation and disengagement hypotheses allowed us to extract their current situation. Specifically, the OAs were using technologies following the stimulation hypothesis during the pandemic because they wanted technologies to connect them with people who live apart, while they were using technologies following the disengagement hypothesis after the pandemic because they wanted to help themselves (Table 1). This is the strong evidence that the OAs are becoming in an increasingly “digital excluded” (Age UK, 2018) situation.

As described by the stimulation and disengagement hypothesis, technology can be designed and used to disengage people from others or stimulate social interaction. For example, one participatory design study investigated a virtual-reality artifact to help OAs reminisce. This technology promoted OAs’ wellbeing (Veldmeijer et al., 2020) and could be used either for disengagement (if the OAs talk only to the machine) or stimulation (if the OAs use the machine to help them reminisce to family or friends). To help OAs connect with others, it is important to develop technology that promotes the latter. Prior work shows that OAs can be socially connected in three ways: to other people, to a neighborhood, and to a society (ten Bruggencate et al., 2019). That study recommended ways technology can connect OAs such as by helping them find ways to volunteer (ten Bruggencate et al., 2019). We recommend that researchers bring these themes of disengagement and stimulation to the forefront when conducting PD workshops to help understand the situation of socially vulnerable peoples like OAs and to motivate the research community in developing the appropriate technology to help them and stimulate social connection.

### 5.2 Limitations

There are some limitations in this study. The first limitation was about the assignments of the participants; that is, no participants experienced both workshops during and after the COVID-19 pandemic. We compared the results of the PD workshops between during and after the pandemic, but there were no overlaps in the participants. This study is not a rigidly prepared longitudinal study to observe the participants’ attitudes change. The second one is the average age of the participants, being middle to older adults, rather than older adults. The average age in the PD workshops in 2021 was 55.4 years old (SD. = 4.17) while the one in 2023 was 57.6 (SD. = 5.12). Even though the 2023 study participants are older, the typical retirement age in Japan is 65–70 years old (70 becoming more normal), so the participants in these PD workshops are active not in their retirement. The third one is the lack of the participants’ detailed information such as academic background or experiences with technologies. These information can be an analysis factor to deeply understand the participants’ answers or justifications, so our consecutive studies should be designed to correct these information.

We ran these studies on one group of participants: OAs in Japan. Of course, other populations will be different and are important to study. We encourage researchers to perform similar PD workshops in other countries and with other age groups; we will therefore share all our materials in detail including the coding rubric, to increase our community’s understanding of how needs of various populations are similar and how they differ.

Someone will point out that the COVID-19 pandemic is not the only differences between the PD workshops in 2021 and in 2023 or that this seems to be confusing correlation with causation and implying that COVID-19 is causing some of the changes or a digital technology revolution. However, the effects of the pandemic on our lives are tremendous, and it facilitated the rapid introduction of various kinds of technologies including the generative AIs, or telecommunication tools. So we would like to argue that the other factors that seems to be independent from the pandemic should not be considered independently but be a part of side-effects of the pandemic. Actually some studies (Grinin et al., 2022; Tarhini et al., 2022) supported these arguments, so we believe that the results of the two PD workshops might reflect the participants’ understanding of the COVID situation “during” and “after” the pandemic.

## 6 Conclusion and future work

By means of the participatory design (PD) workshops during and after the pandemic, we were able to clarify what social issues and difficulties the Older Adults (OAs) faced during and after the pandemic and what they wanted for robot technology to resolve those issue. Specifically, during the pandemic, their most serious issues were communicating with distant family members, and they had dominant altruistic opinions such as wanting to help their distant family members via technology; this follows Nowland et al.‘s stimulation hypothesis in their model of the relationship between social Internet use and loneliness (Nowland et al., 2018). However, after the pandemic, their issues shifted from the others to themselves; they wanted technology to take care of themselves in anxious and lonely situations. Social isolation and loneliness have been the major social issues of OAs before the pandemic. After the special circumstances of the pandemic had passed, many people felt that society was returning to the way it was before the pandemic in both positive and negative ways. A new development was the rapid introduction of technology in the midst of the pandemic (digital transformation), which made the OAs more anxious and uneasy, and may increase their social isolation and loneliness more. Therefore, this follows the disengagement hypothesis.

At the beginning of the paper, the following two research questions were presented:

• RQ1: How did OAs’ needs for robot technology change from during to after the pandemic?
• RQ2: How were OAs’ attitudes toward such technology during and after the pandemic explained by the stimulation and/or disengagement hypothesis?

Now we could answer the two research questions as follows:

• Answer to RQ1: OAs changed their needs for robot technology from “helping the others who live apart” to “helping themselves.”
• Answer to RQ2: OAs’ attitudes toward such technology during the pandemic could be explained mostly by the stimulation hypothesis, while these after the pandemic could be explained mostly by the disengagement hypothesis. This analysis clearly reflects the current OAs’ situation that the OAs are becoming digital excluded after the pandemic.

For older adults to make effective use of these effective technologies, it is important for society as a whole to confront the social issues faced by OAs by learning about and designing technology to meet their needs, such as through future PD workshops like our own and participatory sessions related to specific technologies like self-checkouts. We then argue that conducting these PD workshops routinely not only in Japan and US but also in the other countries or conducting the longitudinal PD workshops with recruiting the same participants will help the research community to understand the situation of socially vulnerable peoples like OAs and to motivate the community in developing the appropriate technology to help them.

## Data Availability

The raw data supporting the conclusion of this article will be made available by the authors, without undue reservation.

## Footnotes

1
https://www.cdc.gov/coronavirus/2019-ncov/your-health/end-of-phe.html

2 The World Health Organization (WHO) and the United Nations define an “aging society” as one in which more than 7% of the population is 65 years or older, an “aged society” as a society in which more than 14% of the population is 65 years or older, and a “super-aged society” as a society in which more than 21% of the population is 65 years or older (Tahara, 2016).

3
https://line.me/en

4
https://zoom.us

5
https://www.microsoft.com/en-us/microsoft-teams/group-chat-software

6
https://chat.openai.com

7 Visualizations of four PD sessions (Supplemetary Figures S1–4) were shown in appendix as Supplemetary Material.

## Supplementary material

Supplementary Figures S1-S4 are visualizations of the PD sessions. These figures provide an overview of flow during the PD sessions. The red, green, and blue shading in the figures call out the top 3 concepts (Interactive robot, Proxy robot, and Assistant robot) across 4 PD sessions, and arrows show how the concept evolved. The black arrows indicate concepts that participants created and then carried forward their idea for development. The gray arrows indicate that another participant either further developed the concept or had a similar concept.

SUPPLEMENTARY FIGURE S1
The first PD session was held on June 17, 2023 and had 3 participants.

SUPPLEMENTARY FIGURE S2
The second PD session was held on June 17, 2023 and had 3 participants.

SUPPLEMENTARY FIGURE S3
The third PD session was held on June 18, 2023 and had 3 participants.

SUPPLEMENTARY FIGURE S4
The fourth PD session was held on June 18, 2023 and had 3 participants.
