# Supplementary figures and images for: How did COVID-19 pandemic affect the older adults’ needs for robot technologies in Japan?: comparison of participatory design workshops during versus after the COVID-19 pandemic
Source: Front Robot AI. 2024 Jun 4;11:1363243. doi: 10.3389/frobt.2024.1363243 (PMC11184464; doi:10.3389/frobt.2024.1363243)

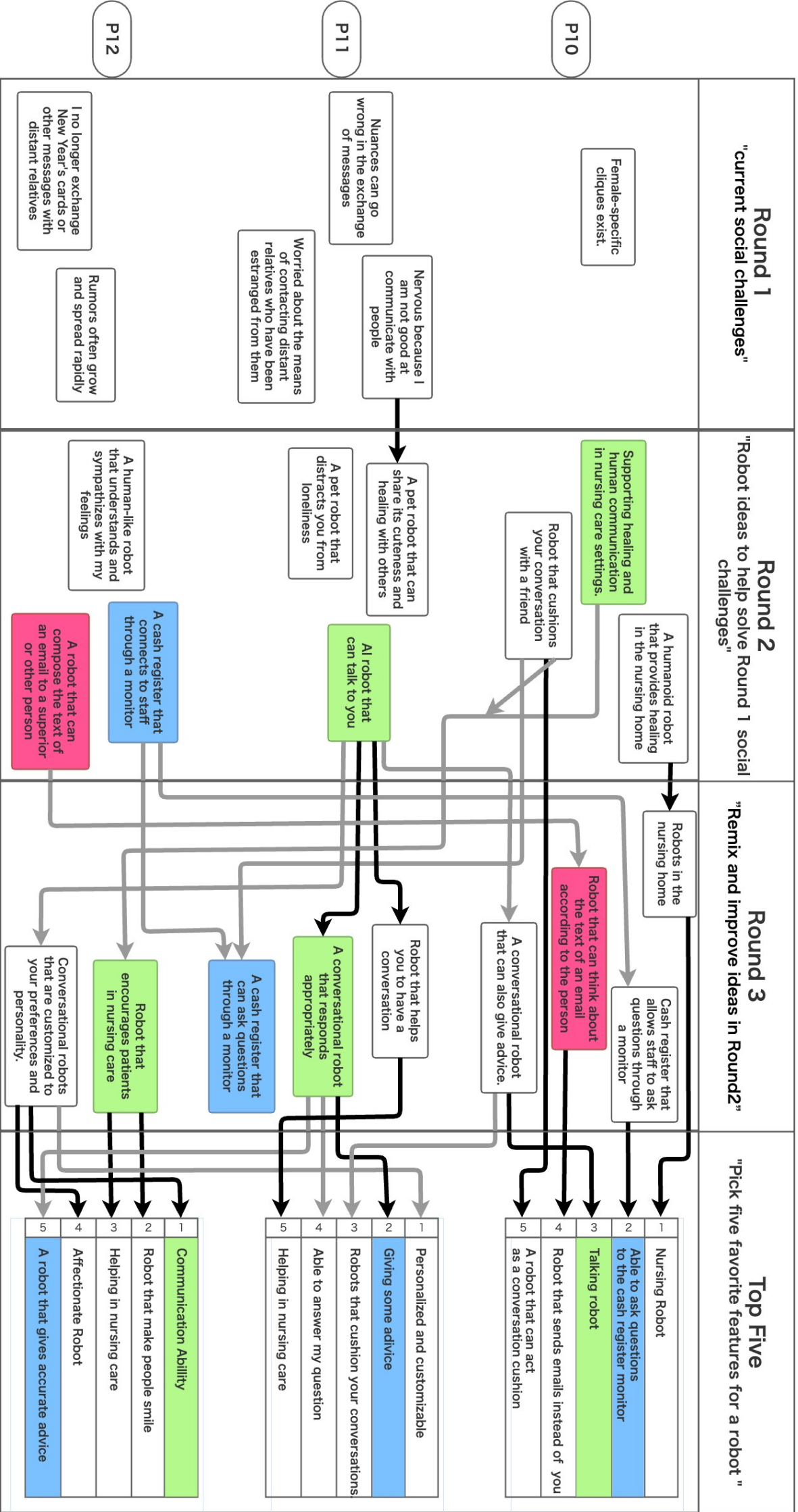

Supplement: Supplementary file 1 [file Image4.pdf]

Interactive Robot  
Proxy Robot  
Assistance Robot

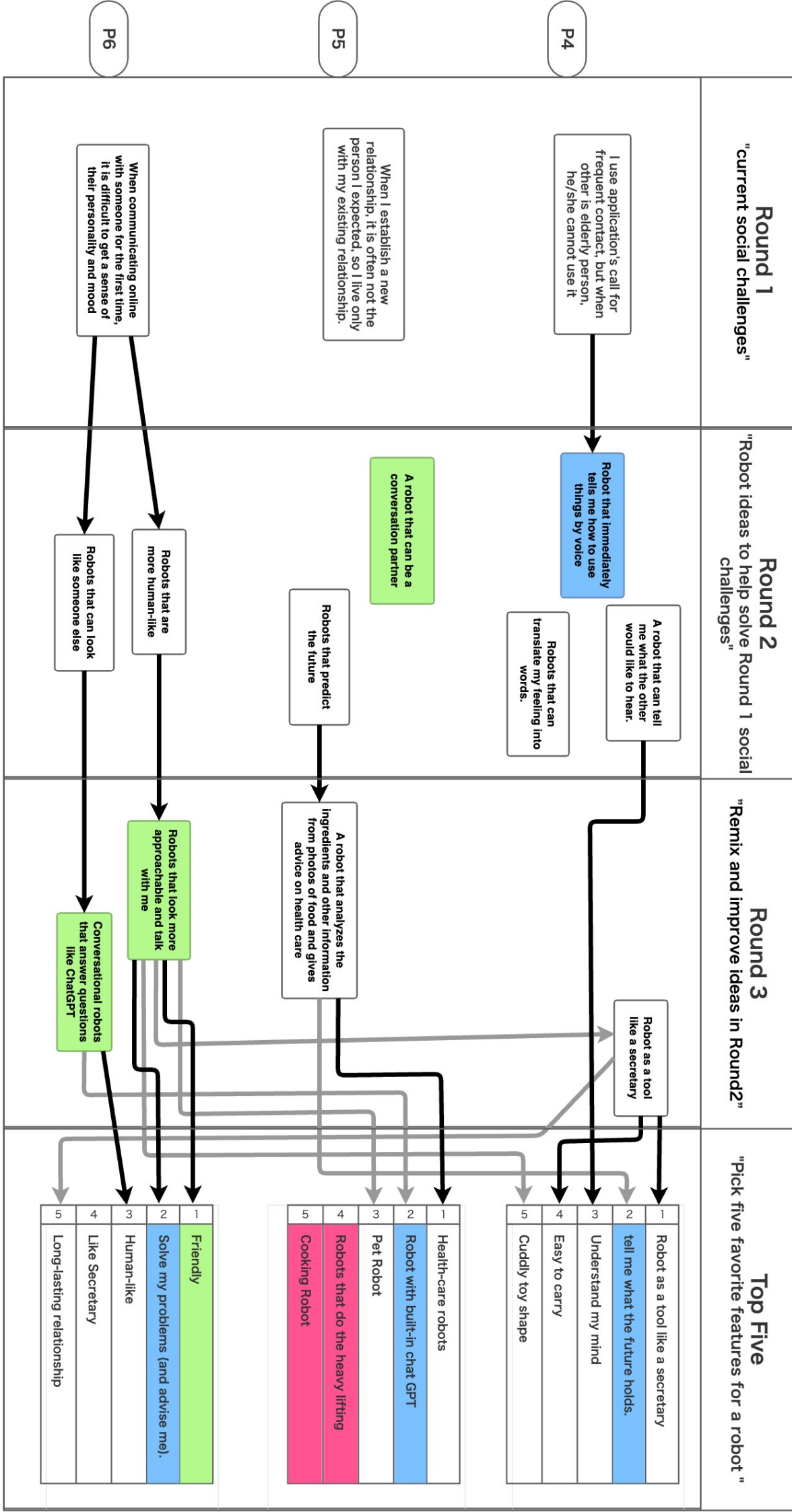

Supplement: Supplementary file 2 [file Image2.pdf]

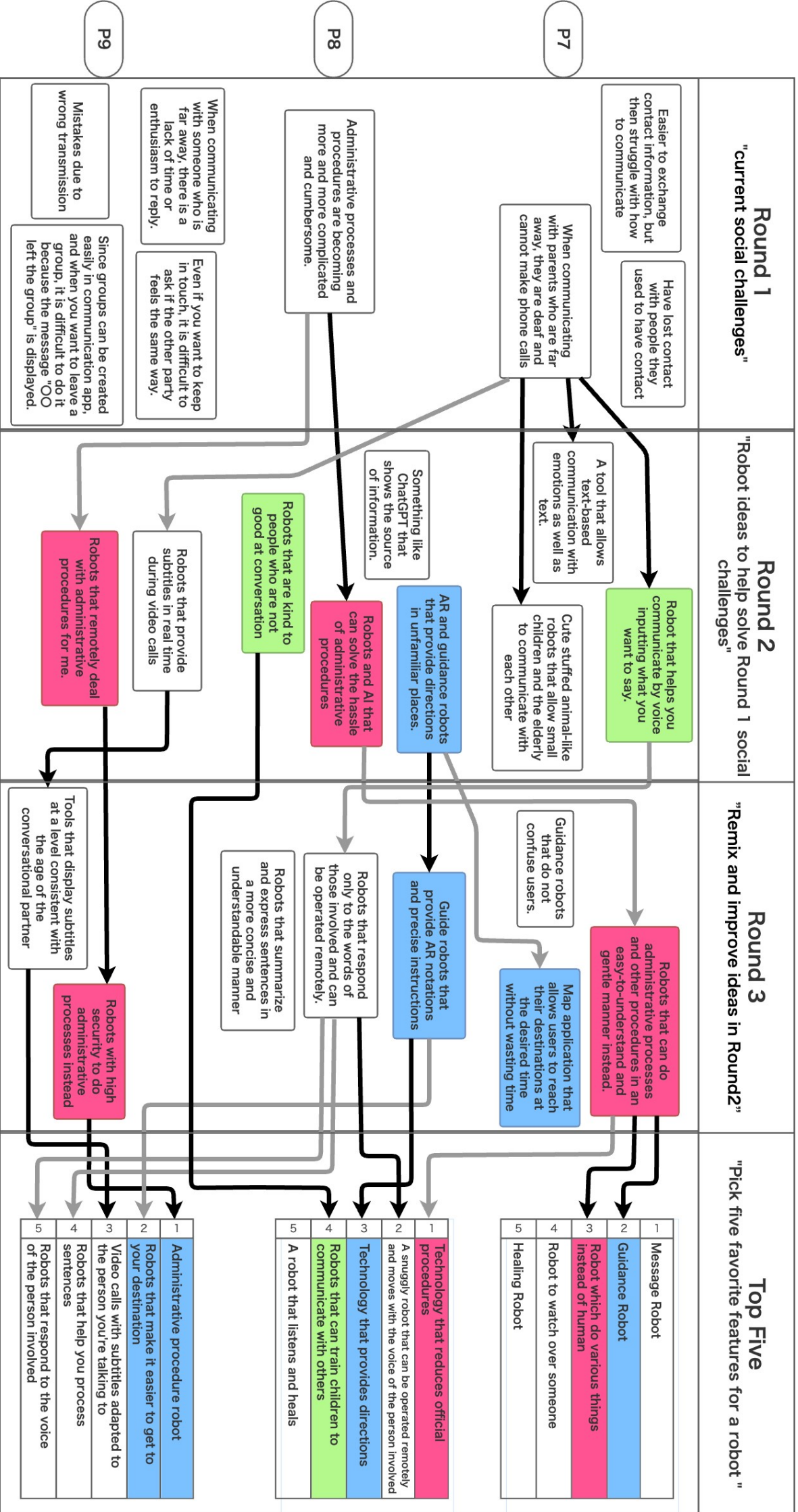

Supplement: Supplementary file 3 [file Image3.pdf]

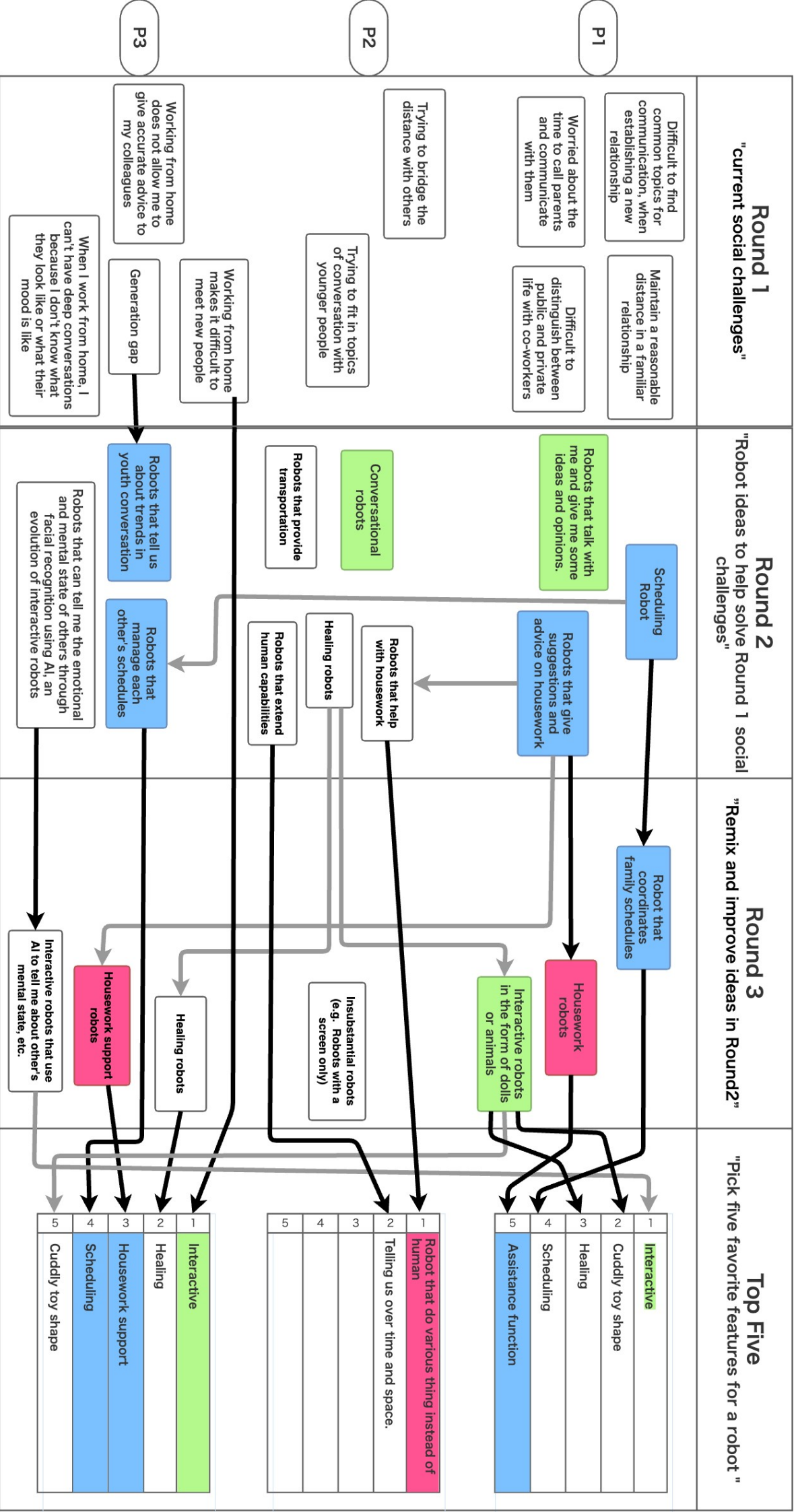

Supplement: Supplementary file 4 [file Image1.pdf]
